# Supplementary material for: Knowledge exchange in the implementation of National Environmental Programmes (NEPs) in China: A complex picture
Source: PLoS One. 2023 Jul 13;18(7):e0288641. doi: 10.1371/journal.pone.0288641 (PMC10343062; doi:10.1371/journal.pone.0288641)
Supplement: S1 Appendix — (PDF) [file pone.0288641.s001.pdf]

Topics with scientists

Part 1 General assessment on the local environment

Q1: Have you observed changes in the local biophysical environment of the station and its surrounding areas in recent years? (How wind, precipitation, soil, plant, sandstorm changes in recent years?)

Q2: What factors do you think are contributing to the changes? (Skip it if "no" with Q1)

Part 2 Academic background

Q1: Which research fields are you in?

Q2: How long have you been engaging in them?

Q3: Who do you think can benefit from your research knowledge? and why?

Q4: Whom would you like to share your knowledge with (the governments, working institutions, peer scientists, or local communities, e.g., grassroot implementers, farmers, entrepreneurs? Why?

Q5: Which of the following groups to you engage with the most and the least to communicate your findings, understanding, or professional opinions?

A. Academic setting (journals, conferences, workshops…)
B. Social media
C. Community face-to-face talks
D. Others, please specify ____

Q6: Why the choice(s)?

Part 3 Local environmental risk assessment

Q1: Which NEPs have been in place in the area around the station since 2000?

Q2: Have the NEPs had impacts on local biophysical environment, and if so, what kinds of impacts?

Q3: How do you see the condition of local natural resources to be heading in the future? Are they degrading or improving or staying the same? What makes you think this?

Part 4 Knowledge communication

Q1: Have you ever been engaged in pilot demonstration projects at the station?

Q2: Do you think the pilot demonstration projects can play a role in combating desertification? If yes, could you please give some examples?

Q3: Do you consider grassroot implementers and local farmers have important local

knowledge that can help the NEPs? Can you give some examples if so?

Q4: Do you consider you have sufficient opportunity in your work to interact with frontline scientists, grassroot implementers and local farmers? If yes, have you been learning about their perspectives or have they been learning about your scientific knowledge, or both?

Q5: What do you think of the communication with farmers? What do you think of communication with local governments, frontline scientists, and grassroot implementers?（easy/difficult? Necessity? In which respects?）

Q6: What mechanisms and approaches do you find most helpful in interacting with non-researchers to share your knowledge?

Q7: Do you think scientists have been actively engaged during the process of formulating NEPs? If yes, in which form? If no, why?

Part 5 Perspectives

Q1. Based on your findings, what would be your first suggestion to local implementers? Why?

Q2: What would be your first suggestion to local farmers based on your professional understanding? Why?

Q3: Given your research topics, what would you like to know most from them?

Q4: What are the 3 most important topics you believe should be studied for combating desertification in China in the future?
