# Supplementary material for: Knowledge exchange in the implementation of National Environmental Programmes (NEPs) in China: A complex picture
Source: PLoS One. 2023 Jul 13;18(7):e0288641. doi: 10.1371/journal.pone.0288641 (PMC10343062; doi:10.1371/journal.pone.0288641)
Supplement: S2 Appendix — (PDF) [file pone.0288641.s002.pdf]

Topics with grassroot implementers

Part 1 General Assessment of the NEP

Q1: What kind of changes have happened since the implementation of the NEP?

Q2: Do you think the NEP has achieved its goals?

Part 2 Engagement with the implementation procedures

Q1: How long have you been engaging in NEP implementation?

Q2: Did you have any support/training when you began?

Q3: What were your main tasks?

Q4: Looking back, what was the most challenging part of your work?

Part 3 Implementation Assessment

Q1: What were the major measures in the implementation process?

Q2: Which measures were easy to implement, and which were difficult? And why?

Q3: Do you think all the measures have served their purposes well?

Part 4 Engagement with local community

Q1: What impacts do you think the program has had on local farmers?

Q2: What mechanisms do you use to interact with them?

Q3: How frequently do you interact with them?

Q4: How would you comment on the interactions with local farmers?

Part 5 Knowledge communication

Q1: What were difficult questions to answer during the implementation? And why?

Q2: Did you ever ask advice from scientists or local farmers?

Q3: What kind of knowledge do you think they need? Why?

Q4: What would make you share your knowledge with them? Why?

Part 6 Perspectives

Q1. Based on your experience, what would be your first suggestion to local farmers? Why?

Q2: Similarly, what would be your first suggestion to scientists? Why?

Q3: Given your work demands, what would you like to know most from them?

Q4: If you were to implement another NEP in the future, what would you do differently?
