# Supplementary material for: Knowledge exchange in the implementation of National Environmental Programmes (NEPs) in China: A complex picture
Source: PLoS One. 2023 Jul 13;18(7):e0288641. doi: 10.1371/journal.pone.0288641 (PMC10343062; doi:10.1371/journal.pone.0288641)
Supplement: S3 Appendix — (PDF) [file pone.0288641.s003.pdf]

Questionnaire survey

(Explain the surveyed project information sheet. Ask for their verbal consent. Each survey will take approximately 30-45 mins)

Section 1: General

1	name of head of household	Wang			
2	Gender of head of household	M		F	
3	Estimated age	18-30	31-50	50-65	65+
4	Number of people in household	1-2	3-5	6-8	9+

Section 2 : Land and Land use

5	How long ago were you allocated land (years)?	<5	6-10	11-15	16-20	21-40	41 +	DK
6	How much land was you allocated (ha/mu)?							
7a	How much land do you have now (ha/mu)? （							
7b	What types of land are they?	Slope land	Irrigation land	Grass land	Other			
7c	Did you have sufficient land to provide food for your family last year?	Y	N					
7d	When was the last time your harvest was lower than you hoped for?	(year)	Never	Prefer not to say	Other			
7e	Why do you think the yields were lower than you hoped?	Frost	Drought	Lack of manure/fertilizer	Disease	Not weeding	Other	
7f	How did you get food?	Go to buy	Ask government	Ask family for help	Ask neighbors	Prefer not to say	Other	

Section 3: Arable land

8a	Which crops did you grow last year?	Maize	Rice	Wheat	Millet	Other	None
8b	Which others?	Beans	Cabbages	Cucumbers	Cotton	Green-leaf vegetables	Other(s) Tomatoes
9a	Do you sell the crop (s)?	Y	N				
9b	If yes, what percentage did you sell last year?	1-29%	30-49%	50-69%	70-90%	Other	
9c	Where do you sell them?	Village	Town	County	Other		

10	Has your yield increased in the past 5 years?	Y	N	Varies	DK
11	Has your yield increased in the past 10 years?	Y	N	Varies	DK

12	Do you apply fertilisers?	Y	N	N/A
13	Do you apply manure?	Y	N	N/A

14	How do you plough your land?	Oxen	Tractor	Both	Other	N/A

15a	Did you fallow your land? If so, how long for?	Y for ___ years	N

15b	Why?	Restore soil fertility	Stop pests	DK	Tradition	Others

Section 4: Fuel

16	What kind of fuels do you use for daily life? (tick all that apply)	Coal	Gas	Electricity	Straw	Solar	Wind	Charcoal	Fuelwood	Other

17	Do you use wood grown on your land as fuel?	Y	N

18	Has time spent collecting wood increased, decreased, or stayed the same over the last 5 years?	Inc	Dec	Same	DK	Other
19	Has access to wood increased or decreased over the last 10 years?	Inc	Dec	Same	DK	Other

20a	If access increased, why?	More trees	Changed policy	DK	Other

20b	If access decreased, why?	No trees near	No new trees	Policy ban	DK	Other

Section 5: Natural resources

21	Did you harvest any other natural resources last year?	Y	N (if no, go to section 6)

22a	What did you harvest?	Grass	Fruits	Both	Raw herbal medicine	Other

22b	During which months did you harvest grass?	Jul	Aug	Sept	Other

22a	Which fruits do you harvest?	Apple	Pear	Peach	Strawberry	Date	grape	Watermelon	Other

22b	When harvest raw herbal	March-April	May-	Aug -Oct	other

	medicine		Jul		

22c	When harvests other?	March-May	Jun-Aug	Step-Oct	Nov-Feb
23	Who was allocated the land you harvest the resource from?	Government	Collective	Own	Other
24a	Do you sell the resource?		Y		N
24b	If yes, where?	Village	Town	County	Along the road	Other
24c	How do you get there?	Walk	Bus	Collected	other
25	Do you sell the resources in their raw state?		Y		N
26	Has access to wild resources increasing or decreased in the last 5 years?	Inc	Dec	Same	Varies	DK
27	Has time spent harvesting wild resource increased or decreased in the last 10 years?	Inc	Dec	Same	Varies	DK

Section 6: Livestock (NO)

28	Do you keep poultry?		Y		N	
29a	How many poultry?	＜10	10-20	21-30	31-40	41+
29b	Do you keep cattle?		Y		N	
29c	How many cattle?	1-2	3-5	6-10	11-20	21+
29d	Do you keep goats?		Y		N	
29e	How many goats?	＜10	10-20	21-30	31-40	41+
29f	Do you keep sheep?		Y		N	
29g	How many sheep?（有多少只？）	＜10	10-20	21-30	31-40	41+
29h	Do you keep any other animals?				Y	N
29i	How many?	1-2	3-5	6-10	11-20	21+
30	Have cattle/sheep/goat numbers changed over the last 5 years?			Inc	Dec	Same
31a	Why the increase?			Bought	Bred	Other
31b	Why the decrease?			Sold	Died	Other
32	Why do you keep animals?	Food	Bank	F&B	Tradition	Other

Section 7: Income

33	Main sources of cash income?	Arable sale	Job (if ticked, cont.' with the following questions)	Seasonal job (if ticked, cont.' with the following questions)	Other	None

34a	Where was the job/seasonal job found?	Town	County	Nearby cities	Big cities	Other

34b	What are the most likely sectors to find a job/seasonable jobs?	Construction sites	Factories	Restaurants	Delivery business	Other

34c	Is it easy to find a job/seasonal job?	Y	N

34d	What are main reasons that motivated you to find the job?	Support family	Easy money	Seasonal arable activity pause	Friend invitation	Other

35	Are you going to move your family to the place where you work?	Most likely	likely	DK	unlikely	Very unlikely

36a	What would make you decide to move?	Children's education	Medical services	More money	Decreasing harvest	Degrading environment	Other

36b	What would make you decide to stay?	Harvest well	Improved environment	Improved infrastructure	Can't find a job	Tradition	Other

Section 8: Environmental change

37	Is the grazing pasture quality/soil fertility good at the moment?	Y	N	DK

38a	Why is it good?	Few sheep/goats/cattle	Project improved it	DK	Enough land	Use rotational grazing/fallowing

38b	Why bad?	Little rain	Strong and constant wind	DK	Sheep/goats/cattle track	Slope	Lack of mgt	Other

39a	How do you recognize that it is good?	Fat cattle/ sheep/ goats	Fat cattle /goats /sheep and good grasses	Good harvest	Lots/ good grass	Less sandstorms	DK	Cattle/ sheep/ goats live longer

39b	How do you recognize that it is bad? (	Poor harvest	Short grass	Lots cattle/ sheep/ goats	Bare ground & gullies & rocks	Thin/dead cattle/ sheep/ goats	Cattle/ sheep/ goats walk away	Dry grass	DK

40a	Has the pasture quality/soil fertility changed in the last 5 years?	Worse	Better	Same	DK

40b	Has the pasture quality/soil fertility changed in the last 10 years?	Worse	Better	Same	DK

41a	Have the bush and grass species present on your land changed in the last 5 years?	Y	N

41b	Nature of change?	Planting	Air seeding	Less G	Short	DK	More	Longer

42a	Has the amount of bare ground on your land changed in the last 5 years?	Inc	Dec	Same
42b	Has the amount of bare ground on your land changed in the last 10 years?	Inc	Dec	Same

43	Have you ever seen the soil on your land washed away by rain?	Y	N

44	What do you think causes soil erosion?	Heavy rain	Cattle/ sheep/ goats	slopes	DK	Others

45	Is soil erosion a problem for you?	Y	N

46	How serious is the problem out of 5, with 1=no problem, 2=slight erosion, 3=moderate erosion, 4=severe erosion, 5=extreme erosion?	1	2	3	4	5

Section 9: About the NEPs

47a	Do you carry out any activities to conserve soil on your land?	Y	N

47b	If yes, what kind of activities?	Furrows	Grass strips	Terracing	Strips/plant trees/fill gullies	Others

47c	Why do you use this/these activities?	Tradition	Told	Cheap	Easy upkeep	DK/other

48	How successful are strips/furrows… out of 3, 1=very successful, 2=successful, 3=not successful	1	2	3

49	Have you heard of the concept of desertification?	Y	N

50	If yes, where from?	Grassroot implementers	Social media	NGO	TV broadcasting	Others

51	Have you heard of TNSP/GGP?	Y	N, go to 53

52a	Can you describe what are major measures with TNSP/GGP?	A. Cash subsidies for retiring slope lands
		B. Grain subsidies for retiring slope lands
		C. Planting trees on slope lands
		D. Planting grass on slope lands
		E. Confined cattle/sheep/goat raising
		F. Seasonal grazing
		G. Others, please specify

52b	Which measures do you prefer? (select one or more of the above choices)	

52c	Why?	Extra income	Extra grain	Good environment	More job opportunities	Other

53	Can you tell me if you think the following activities are very important, important or not important?			
Activity	VI	I	NI	DK
Making people aware of who to approach with problems relating to damaged land				
Educate people about environment problems they might face				
Encourage people to join in with the community activities to help the environment				
Encourage people with different resources to deal with desertification with different ways				
Enhance the role of scientists in policy making process				
Mend damaged land				

Help the government to make a set of rules about the use of trees				
Improve research and technology for farming and help reduce damage to the land				
Develop other fuels for people to use				
Improve the ways in which livestock are managed				
Develop plans to reduce the effects of drought and poverty				
Improve local infrastructures and community services (transport, school, hospitals)				
Help the government to create a land use plan				
Help the government to create a settlement and resettlement policy				
Control population growth				
Others				

Section 10 Knowledge communication

54a	Have you got any information about the NEP when it was to be implemented on your land?	If Yes, what kind?	N	Others

54b	If yes, where from?	Grassroot implementers	Social media	NGO	TV broadcasting	Others

54c	If yes, do you think it is helpful with the understanding of the implementation on the land?	Y	N	Other

54d	What kind of information you would like to know about the NEP on your land?	Effects the NEP will have	Actions to be taken on the land	Supportive measures (e.g., sapling supply, mechanic availability etc)	Compensations	Other

54e	From whom do you expect to have the information?	Local government	Grassroot implementers	Scientists	Village head	Other

54f	Why?	More accurate and reliable	Easy understanding	Amicable attitude	Often being available	Other

55a	Have you ever been involved in demonstration visits to the station?	Y	N

55b	If yes, how do you think of them?	Very helpful	Helpful	Not relevant	Not helpful	Other

56	What kind of information you would like to have more? （	(Local) Environmental	(Local) Educational	(Local) Entertainment	(Local) Employment	Local policy	Other

57	How do you usually get the information?	Friends talks	Social media	NGO	TV broadcasting	Other

58	Have you considered sharing your knowledge with grassroot implementers and scientists?	Y	N	Others

59	Why?	Cannot meet them	Have no opportunity to talk	Nothing to share	They know more	Other

60	What is your greatest fear for the future from a farming perspective?
61	Do you have any other comments to make about farming/soil/drought/land?
