# Supplementary material for: Knowledge exchange in the implementation of National Environmental Programmes (NEPs) in China: A complex picture
Source: PLoS One. 2023 Jul 13;18(7):e0288641. doi: 10.1371/journal.pone.0288641 (PMC10343062; doi:10.1371/journal.pone.0288641)
Supplement: Attachment — Submitted filename: Review Report for PONE-D-22-30804.docx [file pone.0288641.s004.docx]

Review Report for PONE-D-22-30804: **Knowledge exchange in the implementation of National Environmental Programmes (NEPs) in China: A complex picture**

This MS uses a multi-actor-oriented perspective to explore what and how knowledge has been exchanged during the long-term, multi-phase implementation of three major National Environmental Programmes in China. The study employs a mixed-method analysis, combining qualitative interviews with survey data, examined through thematic content analysis and descriptive statistics.

The research findings allow the authors to argue for the need for the environmental governance system to consider the dynamic social contexts and socio-economic needs, adopting an adaptive management approach with more effective knowledge exchange processes and mechanisms as the prevailing are currently hindered by several evidenced barriers.

**My general comments are as follow:**

A brief conceptual framework is needed (but is currently missing) in the MS. It is important that the authors clarify the concept of Knowledge Exchange (KE) and its application / operationalization within the study context, including clarifying different forms of KE and what KE entails in terms of processes or activities.

Despite having conducted a survey, less quantitative analysis (limited to descriptive statistics) is presented in the MS.

There is scope for more discussion about the degree of KE and the adequacy of its forms in the studied context to support effective KE – training/knowledge transfer, communication forms, vertical, hierarchical interactions and relationships, engagement activities?

The MS would benefit from some additional proofreading/copy editing.

**Detailed comments:**

**Introduction:**

Page 2, Paragraph 1 – There is scope here to expand this paragraph to elaborate more on mechanisms to increase knowledge usability and usefulness – see for instance: Dilling and Lemos 2011; Lemos et al. 2012, and many more recent ones.

Page 2, paragraph 2: Why are smallholder farmers and herders qualified as “underrepresented” local communities? Is that particular to the study area? Or are the smallholder farmers and herders studied here from any minority groups/ ethnics?

The sentence “This means that KE can result in mismatch between….” Seems a bit ambiguous. Isn’t KE supposed to align supply and demand of knowledge for increased usability/usage and usefulness?

Page 3, end of paragraph 1: What are the well-established KE mechanisms that you are referring to? Examples??

The Intro ended abruptly right after articulating the research questions and objective. Would be good to introduce the research approach/methodology briefly, a quick summary of the findings, and map the organization/structure of the paper.

**Methodology**

Add a map of the study sites within China, at least showing the provinces or villages/locations. I see an attempt to not disclose the research stations where the study took place without any explanation for doing so.

Page 4, last paragraph at the bottom. Explain what “WeChat” is. It is explained later, on page 5, but I think this should be done here.

Page 5, 2^nd^ paragraph: How did you ensure you were introduced to all critical/relevant potential representatives?

Explain what convenience sampling is.

Page 5, last paragraph: what is the spatial distribution of the 187 survey participants –either in relation to the research stations or the villages/units/areas covered and how many villages/units/areas where covered.

Are the farmers / herders considered as “household” or “individual”? What is the population from which the 187 participants were sampled? What is the proportion covered by the survey?

Did the transcription involve any software?

**Results**

Page 9, bottom last line: “lack of timely KE….” Do you mean communication or dialogue? This is why I suggested unpacking what and how you construed as KE is important up front.

Page 10, last paragraph: Why citing Kong et al. 2021 here and elsewhere within the results section? Are you also introducing secondary data analysis in addition to the thematic content analysis and the survey data analysis?

Page 12, 2^nd^ paragraph: “Some…” can you be more specific – how many?

Page 14, last sentence of the 1^st^ paragraph is a repetition. This was already said on page 12 (bottom paragraph)

Page 17, last 2 paragraphs at the bottom. How are these related to the KE?

**Discussion**

Effectuated??

Page 21: I do not see why contrasting the two sets of evidence. “… and exchange of ….”

See my last general comment above.

**Conclusion**

The conclusion will benefit from more structure. There is need to recall here what the study sets out to examine, briefly summarize the salient findings and the contributions before articulating recommendations and/or additional research needs.
