# Supplementary material for: Knowledge exchange in the implementation of National Environmental Programmes (NEPs) in China: A complex picture
Source: PLoS One. 2023 Jul 13;18(7):e0288641. doi: 10.1371/journal.pone.0288641 (PMC10343062; doi:10.1371/journal.pone.0288641)
Supplement: Attachment — Submitted filename: the rebuttal letter.docx [file pone.0288641.s005.docx]

Dear Editor and Reviewers,

Thank you very much for your comments and suggestions to help improve our paper. Our responses to your feedback are detailed over the following pages. The manuscript has been edited by a native English speaker and now follow the journal’s required style and formatting.

We look forward to your further evaluation of our paper.

Warm regards,

Lindsay, Jouni, and Zheng-Hong

**Table 1 Response to comments from first Reviewer**

| **Comments of the Reviewer** | **Responses of the authors** |
| --- | --- |
| 1. the research methodology is interesting and the paper goes into some detail about the thematic analysis of the interviews and survey details, and whilst the paper states that the results are presented in an actor-oriented strategy, I really think that some more description of the themes and survey results, perhaps in a couple of tables and/or charts, would have benefitted the paper a lot. | 1. Table 5 on page 24 has been prepared to synthesise the themes and results from the actor-oriented observations, containing more details about the engagement patterns. Please also see Tables 3, 4, and 6 on page 16-17, page 18, page 31 which have been added in response to your subsequent comment. |
| 2. The charts in the paper were very basic and could have had more detail and it would have been useful to have reported some simple non-random statistical analysis, such as crosstabes. | 2. Please see Table 3, 4, 6. We have removed some of the earlier, simple charts too. |
| 3. The heading hierarchy in the article is confusing (i.e. page 7), and there are far too many sub-levels of headings, which are not reflected in the text | 3. Headings have been restructured to reduce complexity and are now in line with journal formatting requirements. Considering the complexities of the content, a brief introduction has been added at the beginning of the Results. |
| 4. There is also inconsistent use of text formatting, such as bold, italics, quotation marks etc. | 4. To distinguish direct quotations, we use italics and quotation marks. To align with the journal formatting requirements, the bold phrases have been replaced and distinguished with singular quotation marks instead. |
| 5. Some in-text references (i.e. page 4 but also other pages) give hyperlinks instead of correctly formatted references | 5. All references are now correctly formatted in the journal style. |
| 6. The order given in section 3.1.1.3 regarding the ages of the farmers is given in a strange order (either report these results by ages or percentage in ascending or descending order) | 6. The information has been rearranged in a more sensible order as suggested. Please see Table 3 on page 16. |
| 7. The numbers next to the rather extensive list of quotes are rather confusing, it would have been useful to give a simple code to help with reading, such as "Farmer, 46" or similar | 7. Sentences explaining the codes have been provided. Please see lines 149-160. |

**Table 2 Response to comments from 2^nd^ Reviewer**

| **Comments of the Reviewer** | **Responses of the authors** |
| --- | --- |
| **Introduction:**   1. Page 2, Paragraph 1 – There is scope here to expand this paragraph to elaborate more on mechanisms to increase knowledge usability and usefulness – see for instance: Dilling and Lemos 2011; Lemos et al. 2012, and many more recent ones. 2. Page 2, paragraph 2: Why are smallholder farmers and herders qualified as “underrepresented” local communities? Is that particular to the study area? Or are the smallholder farmers and herders studied here from any minority groups/ ethnics? 3. The sentence “This means that KE can result in mismatch between….” Seems a bit ambiguous. Isn’t KE supposed to align supply and demand of knowledge for increased usability/usage and usefulness? 4. Page 3, end of paragraph 1: What are the well-established KE mechanisms that you are referring to? Examples?? 5. The Intro ended abruptly right after articulating the research questions and objective. Would be good to introduce the research approach/methodology briefly, a quick summary of the findings, and map the organization/structure of the paper. | **Introduction**   1. Please see Line 60-69 where we have expanded on this as requested. 2. We have now explained that smallholder farmers and herders have been less studied than other groups in KE-related research such as policymakers/decision makers in general and that their voices are rarely heard in policy arenas. Please see amendments in line 70-79.   Regarding ethnic groups, Mongolian herders were included in our study, but not because of their ethnicity but because of their occupation as herders. We probably caused confusion by the use of “underrepresented” communities so have amended that now.   3. KE is defined on lines 49-51 where we indicated it refers to “processes that generate, share and/or use knowledge through various methods appropriate to the context, purpose, and participants involved”. Through KE, supply and demand of knowledge can be aligned to increase the usefulness and usability of the knowledge but assumptions are often made about what different groups need in terms of knowledge without checking with those groups. Please see lines 93-96. 4. The ‘mechanisms’ has been deleted and replaced by ‘channels’. Examples are now in lines 111-116. 5. Please see additions in this regard in lines 141-146. |
| **Methodology:**   1. Add a map of the study sites within China, at least showing the provinces or villages/locations. I see an attempt to not disclose the research stations where the study took place without any explanation for doing so. 2. Page 4, last paragraph at the bottom. Explain what “WeChat” is. It is explained later, on page 5, but I think this should be done here. 3. Page 5, 2 nd paragraph: How did you ensure you were introduced to all critical/relevant potential representatives? 4. Explain what convenience sampling is. 5. Page 5, last paragraph: what is the spatial distribution of the 187 survey participants –either in relation to the research stations or the villages/units/areas covered and how many villages/units/areas where covered. 6. Are the farmers / herders considered as “household” or “individual”? What is the population from which the 187 participants were sampled? What is the proportion covered by the survey? 7. Did the transcription involve any software? | **Methodology**:   1. We cannot add a map of the study sites due to the privacy protection we afforded to our participants when they consented to be involved in the study. The study sites included 3 research stations and the surrounding communities. As the stations belong to the national research network and already on the maps, people could easily tell them out once a map of them was presented.   There were substantial worries among them about providing their opinions concerning anything related to government policy, so we need to respect the promises we made by not disclosing their locations. We have added explanation of this at the start of the methodology section. Please see Lines 149-151. For the location, please see line 161-163.   2. Please see Line 195-196 where explanation is now provided. 3. We actually couldn’t ensure all critical/relevant potential representatives could be introduced. To alleviate the disadvantages of the snowball sampling, we first shared our sampling criteria when contacting with the gatekeeper scientists who understood very well about the characteristics of standard sampling. Second, staff and research information were reviewed at the websites prior to the field data collection. We asked the gatekeeper scientists to introduce scientists who had been potentially missed. Third, personal networks were used to expand the number of gatekeeper scientists. This is explained in lines 203-211. 4. Please see line 228-230 where we explain this. 5. Please see line 233-235 where we give the number of villages sampled in relation to each anonymised research station. All villages sampled had been affected by the NEPs. 6. We recorded both household and individual information in the questionnaire. The sample of 187 refers to the number of respondents. When we recorded the impacts and influences of NEPs on the land and livelihoods, the information reflected the situation of each household. We didn’t define specific spatial scopes to choose villages. The population in our survey areas thus were not calculated. This is because in each village, we asked the villagers about the population of their village but we received different estimates and there was no reliable data to use as a basis. Official documents suggest more than 42 million households have been involved in the NEPs during the past 20 years, but this data was not attributed spatially so was also not useful for us to determine a population. A sentence has been added to clarify the issue. Please see line 235-237. 7. The ‘Dictate’ function in Microsoft 365 Word was used to initially transcribe the interviews. However, it only recognised mandarin, i.e., the official Chinese, but not the various dialects so overall the balance was towards manual transcription. Please see line 252-253. |
| **Results**   1. Page 9, bottom last line: “lack of timely KE….” Do you mean communication or dialogue? This is why I suggested unpacking what and how you construed as KE is important up front. 2. Page 10, last paragraph: Why citing Kong et al. 2021 here and elsewhere within the results section? Are you also introducing secondary data analysis in addition to the thematic content analysis and the survey data analysis? 3. Page 12, 2 nd paragraph: “Some…” can you be more specific – how many? 4. Page 14, last sentence of the 1st paragraph is a repetition. This was already said on page 12 (bottom paragraph) 5. Page 17, last 2 paragraphs at the bottom. How are these related to the KE? | **Results**   1. Both communication and dialogue. This has been clarified in line 49-51. 2. It has been removed. 3. Please see line 467. 4. Repetition has now been removed. 5. Relevance to KE has now been added, please see lines 686-687, 688-689, 695-696. |
| **Discussion:**  Effectuated??  Page 21: I do not see why contrasting the two sets of evidence. “… and exchange of ….” See my last general comment above. | **Discussion**   1. “effectuated” is now changed to “insufficiently weighed in” to avoid possible misunderstanding. 2. Totally agree. Please see lines 769-771. |
| **Conclusion:**  The conclusion will benefit from more structure. There is need to recall here what the study sets out to examine, briefly summarize the salient findings and the contributions before articulating recommendations and/or additional research needs. | **Conclusion:**  We have restructured the conclusion in line with your recommendations. Please refer to lines 819-849. |
