# Supplementary material for: Knowledge exchange in the implementation of National Environmental Programmes (NEPs) in China: A complex picture
Source: PLoS One. 2023 Jul 13;18(7):e0288641. doi: 10.1371/journal.pone.0288641 (PMC10343062; doi:10.1371/journal.pone.0288641)
Supplement: Attachment — Submitted filename: the rebuttal letter.docx [file pone.0288641.s006.docx]

Dear Editor and Reviewer,

Thank you very much for the constructive comments and suggestions that helped us to further improve the manuscript. Our responses to your comments and suggestions are outlined in more detail in the following table.

We look forward to your response.

Kind regards,

Zheng-Hong, Lindsay, and Jouni

**Table 1 Response to comments from first reviewer**

| **Comments of the Reviewer** | **Responses of authors** |
| --- | --- |
| 1. P3, l65, specifies climate science, whereas the MS is not overtly about climate science | 1. The word “climate” has been deleted. |
| 2. P4, l75, street-level bureaucrats is not defined (is only done so later on) – should be clarified for an international audience | 2. A definition is now provided on Page 4, line 75-77, citing from Sevä & Jagers (Ref.22). |
| 3. P5, l103, insert ‘The’ before ‘Three’ | 3. Insertion made. P5, line 105. |
| 4. P5, l106, north-western is spelled with different initial capitals in another place in the document | 4. Different spellings were identified, and all are replaced with “north-western”. |
| 5. P5, from lines 111, might be worth a brief description on the levels of government in China | 5. Please see lines 113-117 on Page 5. |
| 6. P6, l 123, ‘afflicted’ is an incorrect term | 6. “Brought about” is now used instead of afflicted. P6, line 126. |
| 7. Methodology, ensure that is written consistently in the past tense (or the same tense throughout) | 7. Inconsistencies have been corrected. What we did in the field and how data was analysed is in the past tense, while explanation of how the findings are presented in this paper is in the present tense. |
| 8. Table 1 is poorly formatted | 8. The table 1 formatting has been corrected to summarize the characteristics of the study sites in a concise way. |
| 9. P9, l201, clarify what is meant by ‘minors’ | 9. Minors refer to people who are under the age of fully legal responsibility; a term used in Research Ethics Approval Form of this project. This has been included on page 9, line 201. |
| 10. Throughout the document, there is inconsistent use of quotes (either single, or double). Some words are also put in quotes unnecessarily (i.e. p33, l715, supply and demand) | 10. Quotes have been checked and all the quotations use double quotation marks to maintain consistency.   We have removed those quotation marks we used previously to indicate academic jargon. We nevertheless retain them for quoted sentences from interviews and from the questionnaire survey categories. |
| 11. P13, l285, should be members | 11. Corrected. P13, line 284. |
| 12. Tables 3, 4 etc, the column with the text ‘person’ is confusing, not sure what it means | 12. The column was meant to represent the number of participants. We have deleted it in Tables 3, 4, and 6 as it might cause more confusion rather than clarification. |
| 13. P17, l379 etc, should Mandarin be capitalised? | 13. Yes. Mandarin is now capitalised. There are 4 corrections made to this. |
| 14. P18, l393, incorrect in-text reference | 14. Corrected. P18, line 392. |
| 15. Table 5, improve the formatting (justify text under the bullet points) | 15. Improvements have been made both to the formatting and wording of Table 5. |
| 16. P26, l563, ideally, do not start a sentence with ‘Actually’ | 16. Now changed to “The reality was”, on Page 26, line 563. |
| 17. A map showing the broad areas (the research stations) would be useful | 17. We cannot add a map of the study sites for confidentiality and anonymity reasons: these were promised to our participants when they consented to be involved in the study. There were substantial concerns among participants about providing views related to government policy. We have added explanation of this at the start of the methodology section. Please see lines 152-154. For the location, please see lines 163-165. |
